# The recent changes in Mitochondrial DNA Part B-Resources (Part B)

**DOI:** 10.1080/23802359.2020.1763015

**Published:** 2020-05-13

**Authors:** Chang Liu

**Affiliations:** Institute of Medicinal Plant Development, Chinese Academy of Medical Sciences

With the rapid technology development, sequencing the organelle genomes becomes feasible even for small laboratories. Besides, several large-scale genome projects such as the “Earth Biogenome Project,” the “Bioscan Project,” and “1000 Medicinal Plant Genome Project”, have started. We are witnessing an influx of animal mitogenomes, chloroplast genomes, and plant mitochondrial genomes. How can the journal Part B serve the scientific community better in the face of these new developments?

To answer this question, we conducted a bibliometric analysis based on journals indexed in Web of Science during the period of 1980–2019. Using “animal” and “mitochondrial genome” or “mitogenome” as the query terms, we found over 4500 out of 49000 papers published on the scope of Mitochondrial DNA Part A and Part B combined. Using “plant” and “mitochondrial genome” as the query terms, we found 29 of 1400 papers published on Part B. Using “chloroplast genome” or “plastome” as the query terms, we found 1200 of 2250 articles published on Part B. Taking these numbers together; it suggests that Part B is the most productive journal for subjects on “animal mitogenome” and “chloroplast genome,” but not for “plant mitogenome.” Why is this, and what should we do then?

There are three issues we should take into consideration to address these questions. First, the mitogenomes differ significantly across kingdoms. The animal mitogenomes are usually a dozen kilobases long. In contrast, plant mitogenomes can be up to a few megabases long. It can be easy to describe an animal mitogenome in 500 words. Still, it is pretty tight to describe a chloroplast genome or plant mitogenome. Second, initially, Part B’s paper usually focused on the assembly, annotation, and phylogenetic classification of the genome. By using software tools such as MEGA, BEAST, MrBayes, etc., it is straight forward now to carry out various analyses such as distance calculation, diversity exploration, and phylogeny construction. The results are usually an integral part of a genome paper, and many authors would like to have these results published along with the genome. Last, the definition of “resources” has been broadened. New experimental protocols, software tools, databases, integrated analytical methods are all great resources. For these reasons, we made several changes. The detailed information can be found at https://www.tandfonline.com/action/authorSubmission?journalCode=tmdn20&page=instructions. Below we outline several of the most important changes.

- For announcement type of paper, the length is increased to 800 words, the limit for one phylogenetic tree remains the same.
- For the rapid communication type of paper, the length is increased to a maximum of 2500 words with no limit for the numbers of tables and figures, and supplementary materials. This change will allow the authors to publish their work with sufficient technical details. Besides, the rapid communication will accept broad-sense resource papers such as those describing software tools and databases, as long as they are within the journal’s scope.
- Following the growth in submissions the journal has seen in recent years, we have further streamlined the peer-review process. Associate Editors will take expanded responsibilities to assess submissions for their suitability for the journal through managing the external review process.
- We have also introduced a new Public Data Policy, requiring mitogenome sequence data to be made publicly available before manuscripts are submitted to the journal. This will ensure that results and data associated with published Part B papers is made available to the public as quickly as possible.
- Our new ‘scoop protection’ policy ensures that authors’ submissions will be protected from the date of their original submission, should another paper on the same mitogenome sequence be published while their paper is under evaluation in the journal.

By implementing these changes, we hope Part B will meet the ever-increasing needs of our authors to have their high-quality work rapidly disseminated.

